# Case Report: Ultrasound-guided dual-target low-temperature plasma ablation for refractory cervicogenic orofacial pain—a report of two cases

**DOI:** 10.3389/fpain.2026.1685344

**Published:** 2026-08-03

**Authors:** Juan Liu, Jianxun Li, Yifan Wang, Xianting Chen, Baishan Wu

**Affiliations:** Beijing Chaoyang Hospital, Capital Medical University, Beijing, China

**Keywords:** C2 dorsal root ganglion, cervicogenic orofacial pain, low-temperature plasma ablation, trigeminocervical complex, vertebral artery v3 segment

## Abstract

**Background:**

Cervicogenic orofacial pain (COFP) is frequently misdiagnosed or undiagnosed, particularly in patients with refractory orofacial pain. When conventional treatments for conditions such as trigeminal neuralgia, dental pathologies, or oncologic pain fail to provide relief, a cervical spondylotic etiology should be considered. We present two cases of COFP secondary to cervical spondylosis successfully managed with minimally invasive intervention.

**Case presentation:**

Both patients present with 3-year histories of unilateral, persistent orofacial pain and dental discomfort. Despite multimodal therapeutic interventions, no significant improvement was observed. Physical examination of the neck and cervical spine MRI both confirmed the diagnosis of cervical spondylosis. After receiving ultrasound-guided low-temperature plasma ablation targeting the C2 dorsal root ganglion and the periarterial region of the vertebral artery V3 segment, both patients experienced immediate and substantial relief from refractory dental and facial pain. Postoperatively, the visual analog scale scores for both patients decreased from 8 to 1. The Jaw Functional Limitation Scale-8 scores improved from 65 to 11 in Patient 1 and from 60 to 8 in Patient 2. Similarly, the Oral Health Impact Profile scores decreased from 45 to 6 in Patient 1 and from 48 to 8 in Patient 2. No recurrence or complications were observed during the 8-month follow-up period.

**Conclusions:**

COFP often manifests with subtle symptoms overlapping trigeminal neuralgia and dental disorders, leading to diagnostic delays. Comprehensive neuroanatomical differential diagnosis is critical. Precision-guided minimally invasive cervical interventions can provide effective symptom control, as demonstrated by this dual-target low-temperature plasma ablation approach.

## Introduction

Orofacial pain syndromes (OFPs) encompass a spectrum of heterogeneous disorders primarily characterized by recurrent pain localized to facial and oral structures (1). According to the International Classification of Orofacial Pain (ICOP), non-dental facial pain (NDFP) is subcategorized into three principal entities: cranial neuralgias, facial pain syndromes resembling primary headache disorders, and idiopathic orofacial pain (1). Clinical evidence indicates that a significant subset of OFP patients exhibit symptoms attributable to cervical spine pathology. Consequently, we introduce “cervicogenic orofacial pain” (COFP) as a descriptive concept. It must be emphasized that COFP is not a validated diagnostic entity but rather a proposed clinical descriptor for a referred pain syndrome stemming from disorders of the cervical spine or associated soft tissues. Hallmark manifestations include unilateral or bilateral dull, aching, or sharp facial and jaw pain, frequently misdiagnosed as temporomandibular disorders, odontogenic pathologies, or trigeminal neuralgia. Concomitant symptoms such as tinnitus, vertigo, or upper limb sensory disturbances may further lead to erroneous diagnoses of otologic or neurovascular conditions, resulting in therapeutic delays. The underlying pathophysiology involves structural cervical abnormalities (e.g., degenerative changes, disc herniation, or joint instability) and cervicomusculofascial dysfunction. Pain referral to orofacial regions occurs via convergent nociceptive processing within the trigeminocervical complex (TCC) (2).

For patients with suspected COFP, a comprehensive, multidisciplinary evaluation integrating neuroanatomical, neurophysiological, and biomechanical perspectives is essential. Characteristic clinical features involve unilateral orofacial pain linked to cervical pathology, confirmed by positive diagnostic cervical blocks, after primary orofacial conditions have been ruled out. Early identification, coupled with precise, targeted intervention, is paramount to substantially enhance quality of life and ensure positive long-term prognosis.

## Case report

Both patients provided written informed consent for case publication. The specific treatment regimens for both patients are delineated in the table Table 1

**Table 1:** The specific treatment regimens for both patients.

Patient	Symptom duration (years)	Treatment regimens
Patient 1	3	Suboptimal efficacy was observed following interventions, including oral ibuprofen, cervical and facial acupuncture, and tooth extraction
Patient 2	3	Inadequate pain relief was achieved with oral analgesics such as an ibuprofen–codeine combination, diclofenac sodium, and acetaminophen/oxycodone, as well as topical analgesic patches

### Case 1

**Figure 1:**
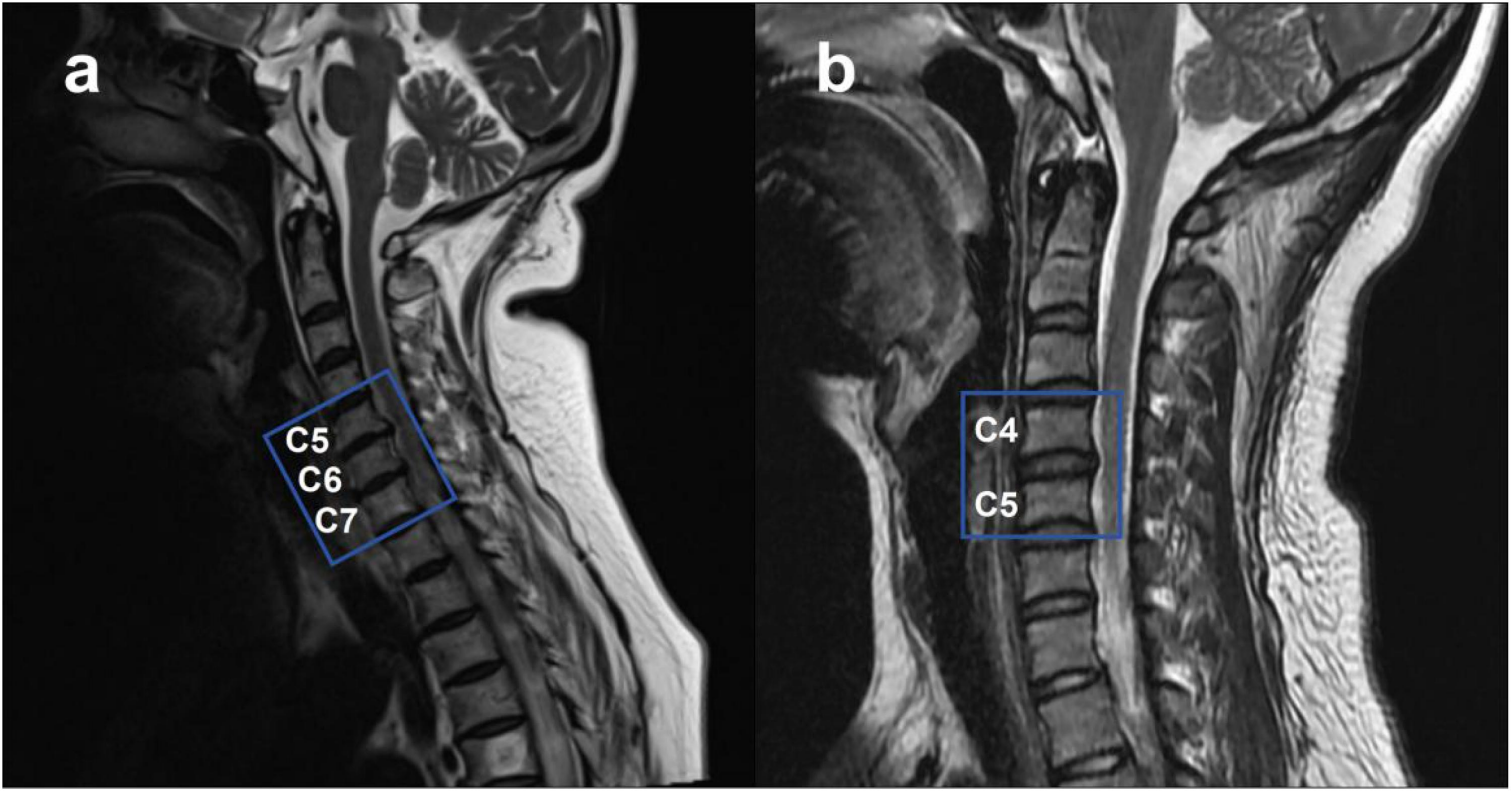
MRI of the cervical spine. (**a**) Cervical spine MRI of Patient 1 demonstrates loss of physiological lordosis, anterior osteophyte formation at the C5–C6 and C6–C7 levels, and disc herniation. (**b**) Cervical spine MRI of Patient 2 shows cervical lordosis and a mild C4–C5 disc herniation.

A 64-year-old man presented with a 3-year history of refractory left-sided orofacial pain. His medical history included left submandibular lymphadenectomy for lymphoma 5 years earlier, followed by tonsillectomy and axillary lymph node dissection for tonsillar carcinoma 3 years ago. Persistent ipsilateral orofacial pain developed postoperatively, refractory to multimodal pharmacotherapy including ibuprofen, diclofenac sodium, and oxycodone. Physical examination demonstrated significant myofascial tenderness in the left suboccipital-cervical region. Cervical MRI revealed loss of physiological lordosis, anterior osteophyte formation, and disc protrusions at C5–C6 and C6–C7 levels (Figure 1a). The findings of both vertebral artery color Doppler ultrasound and transcranial Doppler ultrasound revealed no remarkable abnormalities.

### Case 2

A 35-year-old female reported 3-year history of intractable orofacial pain accompanied by concurrent interscapular discomfort, refractory to comprehensive conservative management. Examination revealed marked tenderness over cervical spinous processes (C3–C7), bilateral paraspinal muscle hypertonicity, trigger points along medial scapular borders, and mildly restricted cervical range of motion (ROM) (rotation <70°). Imaging demonstrated cervical straightening and a mild C4–C5 disc protrusion (Figure 1b). The findings of both vertebral artery color Doppler ultrasound and transcranial Doppler ultrasound revealed no remarkable abnormalities.

#### Surgical procedure

**Figure 2:**
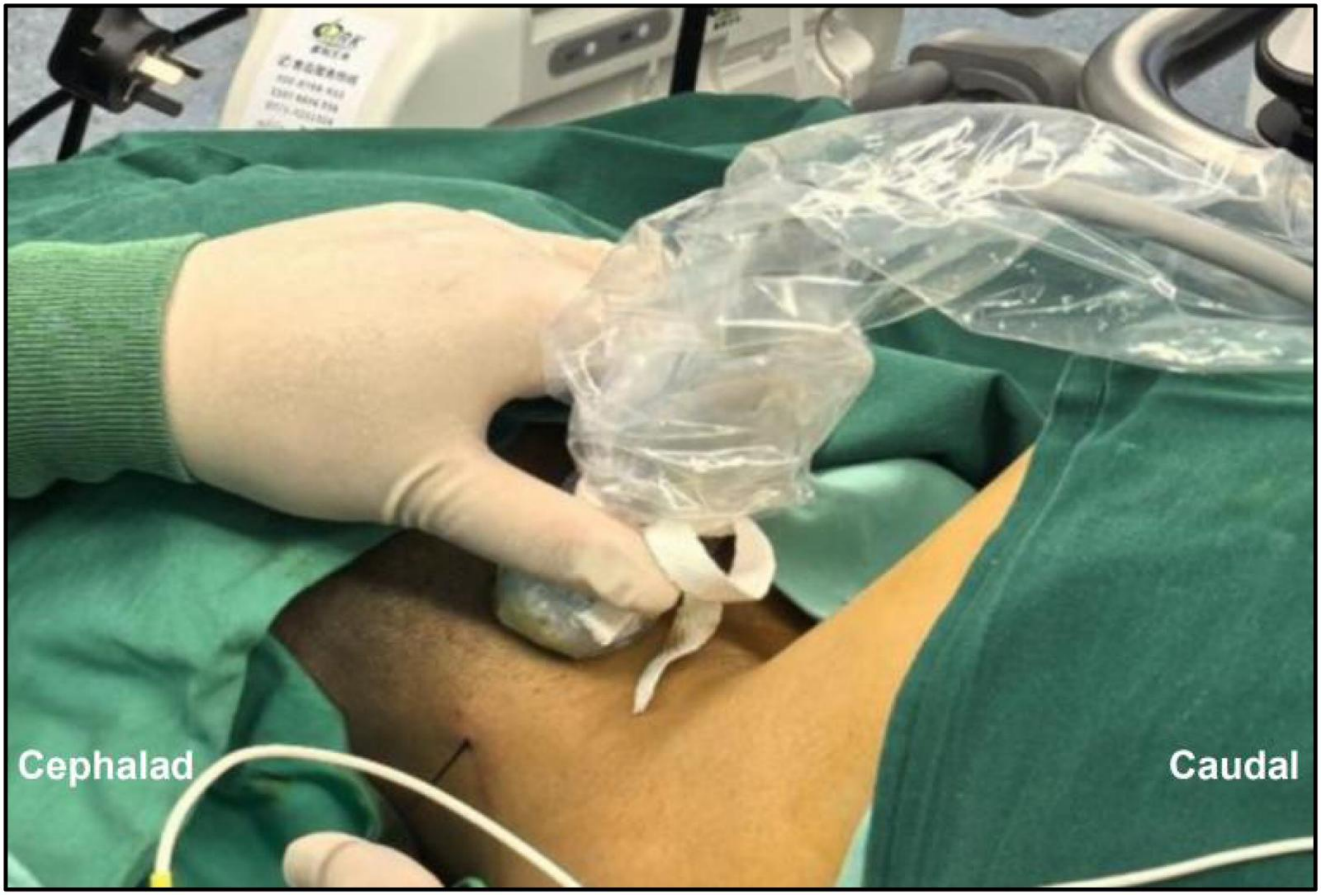
Patient positioning. Lateral decubitus position, affected side uppermost, exposing the posterior cervical region.

**Figure 3:**
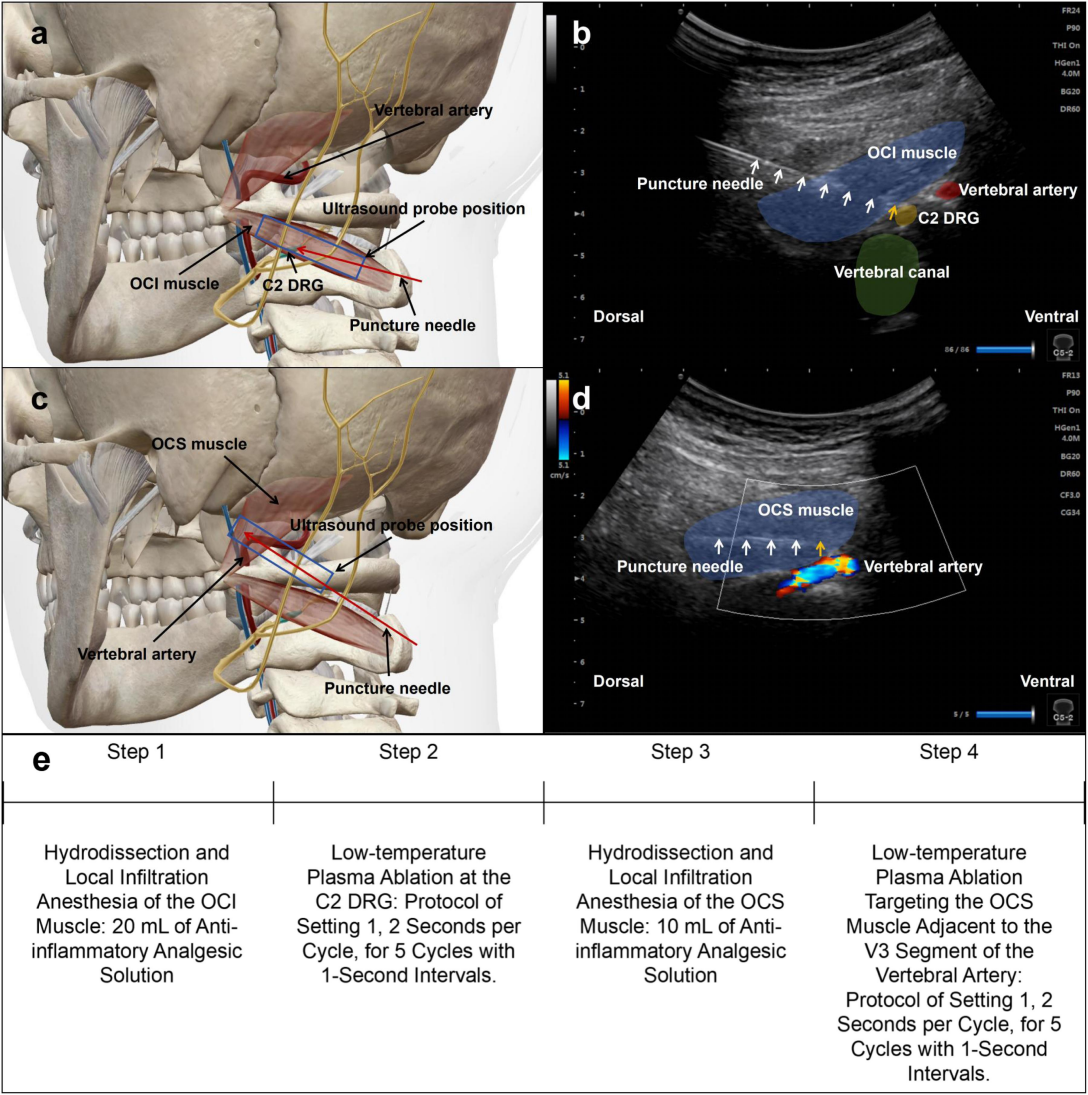
Surgical procedure. (**a**) Anatomical schematic of the C2 DRG puncture approach. (**b**) Ultrasound-guided image of C2 DRG puncture. The needle is advanced obliquely posteromedial to anterolateral from the posterior midline, traversing the inferior OCI muscle, to reach the C2 dorsal root ganglion. (**c**) Anatomical schematic of the needle approach adjacent to the vertebral artery. (**d**) Ultrasound-guided image demonstrating needle placement near the vertebral artery. The needle is advanced obliquely posteromedial to anterolateral from the posterior midline, traversing the OCS muscle, until its tip reaches the surrounding region of the vertebral artery. (**e**) Detailed timeline of the specific surgical procedure.

Preoperatively, a diagnostic blockade of the C2 dorsal root ganglion (DRG) was performed using 3 mL of 0.2% plain lidocaine solution. This intervention provided the patient with more than 70% pain relief lasting approximately 2 h. Based on this positive diagnostic response, minimally invasive surgical treatment was undertaken. Following standard monitoring and intravenous access establishment, the patient was positioned in the lateral decubitus position with the symptomatic side superior. Cervical hyperextension was achieved using neck support to optimize exposure of the target region (Figure 2). A 100-mL anti-inflammatory analgesic solution was prepared, consisting of 1 mL compound betamethasone injection, 200 mg of 2% lidocaine, and 89 mL normal saline, yielding in a final lidocaine concentration of 0.2%. Ultrasonographic localization was performed via a posterior cervical approach. Utilizing a low-frequency curvilinear probe in oblique short-axis view, the long axis of the obliquus capitis inferior (OCI) muscle was identified between the right C1 transverse process and left C2 bifid spinous process—an anatomical landmark termed the “boat sign.” Under real-time Doppler guidance, the vertebral artery and C2 DRG were visualized deep to the OCI muscle, adjacent to the lateral mass of the atlas. The spinal cord pulsations were confirmed medial to the target. After hydrodissection and local infiltration anesthesia targeting the OCI muscle with the anti-inflammatory analgesic solution, an introducer needle was advanced obliquely under continuous sonographic visualization, maintaining safe distance from vascular and neural structures until the C2 DRG was engaged (Figures 3a,b). Following stylet withdrawal, the ablation probe was advanced through the introducer cannula. Sensory stimulation at setting 1 elicited concordant pain reproduction, thereby confirming accurate target localization. Subsequently, low-temperature plasma ablation at setting 1 was delivered to the C2 DRG in 2-s pulses for five cycles, each cycle separated by a 1-s interval.

Subsequently, the ultrasound probe was advanced cephalad to obtain a standardized short-axis view of the obliquus capitis superior (OCS) muscle. Doppler interrogation revealed a characteristic figure-of-eight vascular pattern confirming vertebral artery flow. After hydrodissection and local infiltration anesthesia targeting the OCS muscle with the anti-inflammatory analgesic solution, an introducer needle was advanced obliquely from the posterior midline under real-time sonographic guidance. The trajectory maintained an inferomedial-to-superolateral orientation until the needle tip achieved periarterial positioning without vascular contact (Figures 3c,d). After stylet removal, the ablation probe was deployed. Low-temperature plasma ablation at setting 1 was administered in 2-s pulses for five cycles to the musculofascial tissue surrounding the vertebral artery, each cycle separated by a 1-s interval. Intraprocedural Doppler demonstrated immediate hemodynamic improvement. The assembly was then withdrawn under continuous ultrasound surveillance, followed by sterile dressing application. A detailed timeline of the specific surgical procedure is illustrated in Figure 3e.

## Discussion

COFP is commonly misdiagnosed in clinical practice due to its complex referred pain patterns. In our cases, both patients exhibited unilateral persistent maxillofacial pain characterized by a dull/aching quality—distinct from classical presentations of trigeminal neuralgia (lancinating, paroxysmal pain) (3), odontogenic pain (localized burning sensation with mechanical allodynia) (4), or malignancy-related pain (unremitting severe pain with neuropathic features and sensory disturbances) (5). Through multimodal assessment—including detailed pain phenotyping, physical examination (revealing suboccipital muscle tenderness and restricted cervical ROM), and imaging studies (demonstrating loss of cervical lordosis and disc bulging)—a cervical spondylotic etiology of orofacial pain was definitively established.

**Figure 4:**
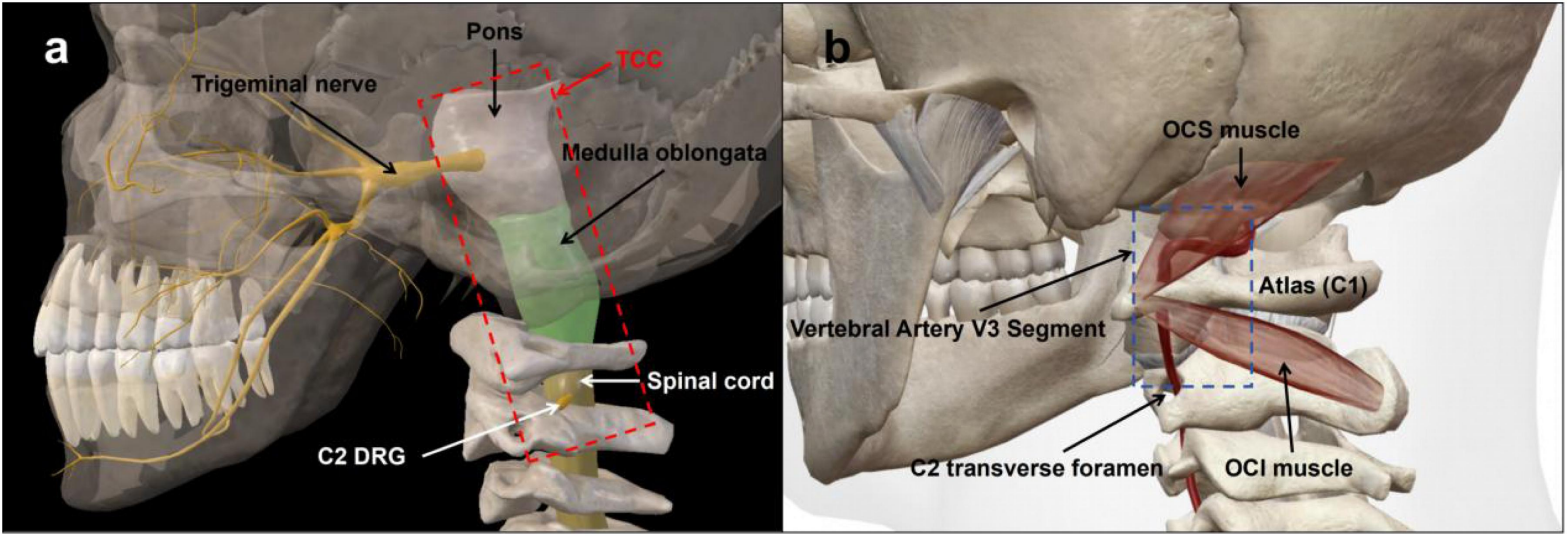
Anatomical schematics of the TCC and vertebral artery V3 segment. (**a**) Schematic representation of the TCC complex: functional unit formed by convergence of caudal trigeminal nucleus and C1–C2 dorsal horn neurons. (**b**) Anatomical course of vertebral artery V3 segment: post-foraminal trajectory after exiting C2 transverse foramen, coursing superior to the atlas (C1). OCI, obliquus capitis inferior; OCS, obliquus capitis superior.

The C2 DRG constitutes a pivotal relay station in COFP transmission. The TCC serves as a neuroanatomical convergence hub, integrating trigeminal afferents with upper cervical (C1–C2) nociceptive inputs (6, 7). This circuitry mediates intersegmental nociceptive signaling from cervical structures to trigeminal second-order neurons (Figure 4a). Pathological cervical spine conditions inducing ectopic discharges in C2 DRG neurons may manifest as referred maxillofacial pain via TCC-mediated neural crosstalk (8, 9). This mechanism elucidates the phenomenon of topographically discordant orofacial pain originating from cervical spondylosis, providing the neuroanatomical rationale for targeted C2 DRG interventions.

The vertebral artery V3 segment traverses a post-foraminal course after emerging from the C2 transverse foramen, ascending superior to the atlas (C1), and penetrating the suboccipital triangle (bounded by the OCI/OCS muscle and the lateral mass of atlas) (10) (Figure 4b). Pathological stiffening or calcific tendinopathy of cervical musculature—particularly the OCS muscle—may induce extrinsic vertebral artery compression, precipitating non-obstructive posterior circulation ischemia (NPCI). Although devoid of structural luminal stenosis, this ischemic milieu exacerbates neuronal hypoxia, potentiating both the initiation and perpetuation of COFP. Targeted low-temperature plasma ablation of compressive myofascial structures significantly improves vertebrobasilar hemodynamics, thereby restoring neurovascular perfusion, abolishing nociceptive signaling, and mitigating recurrence risk. These findings establish a novel pathophysiological framework for COFP management via dual-target neuromodulation.

Low-temperature plasma ablation achieves selective neuromodulation of nociceptive fibers through controlled thermal energy (40–70 °C), inducing transient ion channel dysfunction—primarily affecting voltage-gated sodium channels on C-fibers—that blocks aberrant signal transmission while preserving proprioceptive and motor neuron integrity. This mitigates the risk of irreversible axonal degeneration associated with conventional radiofrequency thermocoagulation (60–90 °C) (11). Moreover, the therapeutic approach in this case series presents technically demanding procedures, wherein real-time ultrasound guidance not only enhances operator proficiency but also mitigates procedure-related complications, while concurrently facilitating diagnostic refinement in orofacial pain disorders.

The preliminary outcomes observed in these two cases suggest the potential clinical feasibility of ultrasound-guided dual-target neuromodulation for suspected COFP. The therapeutic effect may involve a multimodal approach combining mechanistic pain analysis, precise anatomical targeting, and minimally invasive low-temperature plasma ablation. These early findings highlight the need for further investigation in larger cohorts to substantiate the efficacy and mechanisms of this approach. Future investigations must elucidate COFP pathogenesis through advanced neuroimaging and electrophysiological phenotyping while establishing evidence-based indications via prospective, multicenter trials with extended follow-up periods, ultimately enabling the development of personalized treatment algorithms incorporating neurovascular biomarkers. Based on our observations, we propose the descriptive term “cervicogenic orofacial pain” to characterize this clinical presentation, while acknowledging that it remains a non-validated nosological construct. We further hypothesize that non-obstructive posterior circulation ischemia may represent a contributing pathophysiological factor, and suggest that neurovascular interactions could play a role in chronic orofacial pain mechanisms. These exploratory perspectives are offered to stimulate hypothesis-driven research aimed at improving diagnostic and therapeutic approaches for refractory orofacial pain.

## Data Availability

The original contributions presented in the study are included in the article/Supplementary Material; further inquiries can be directed to the corresponding author.
